# Effect of surface treatments on the adhesive properties of metallic brackets on fluorotic enamel

**DOI:** 10.1590/2177-6709.25.4.059-067.oar

**Published:** 2020

**Authors:** Mariana Huilcapi, Ana Armas-Vega, Andres Felipe Millan Cardenas, Lucila Cristina Rodrigues Araujo, Jessica Bedoya Ocampo, Matheus Coelho Bandeca, Fabiana Suelen Figuerêdo de Siqueira, Alessandro Loguercio

**Affiliations:** 1 Escuela Odontologia, Facultad de Ciencias de la Salud “Eugenio Espejo”, Universidad UTE (Quito, Ecuador).; 2 Universidade Ceuma, Departamento de Pós-graduação em Odontologia (São Luis/MA, Brazil).; 3 Universidade Estadual de Ponta Grossa, Departamento de Odontologia Restauradora (Ponta Grossa/PR, Brazil).

**Keywords:** Orthodontics, Sodium hypochlorite, Dental fluorosis, Conversion degree

## Abstract

**Objective:**

To compare the effectiveness of the pretreatment with sandblasting and deproteinization with NaOCl on bond strength (SBS), *in situ* conversion degree (CD) of brackets in fluorotic enamel, and enamel etching pattern.

**Methods:**

A total of 90 non-carious maxillary premolars were used. The teeth were then assigned to six experimental groups according to: enamel surface (sound and fluorotic enamel); surface treatment (Regular etch with 37% phosphoric acid [RE]; 5.2% sodium hypochlorite + phosphoric acid [NaOCl + RE]; sandblasting + phosphoric acid [sandblasting + RE]). After storage in distilled water (37°C/24h), the specimens were tested at 1 mm/min until failure (SBS). Enamel-resin cement interfaces were evaluated for CD using micro-Raman spectroscopy. The enamel-etching pattern was evaluated under a scanning electron microscope. Data from SBS and *in situ* CD values were analyzed using ANOVA two-away and Tukey test (α=0.05). The enamel etching pattern was evaluated only qualitatively.

**Results:**

For sound enamel, RE showed the highest SBS values, when compared to NaOCl + RE and Sandblasting + RE groups (*p*< 0.01). Regarding CD, only NaOCl + RE significantly compromised the mean DC, in comparison with other groups (*p*= 0.002). For fluorotic enamel, the Sandblasting + RE group significantly increased the mean SBS values, in comparison with RE group (*p*= 0.01) and no significant change was observed for CD (*p*> 0.52).

**Conclusions:**

The application of NaOCl or sandblasting associated to phosphoric acid improved the SBS of the brackets in fluorotic enamel without compromising the CD of the resin cement, with improving of enamel interprismatic conditioning.

## RESUMO

**Objetivo:**

Comparar a efetividade do pré-tratamento com jateamento e desproteinização com NaOCl na resistência de união (SBS), grau de conversão *in situ* (GC) dos braquetes metálicos ao esmalte fluorótico e o padrão de condicionamento do esmalte.

**Métodos:**

Foi utilizado um total de 90 pré-molares superiores livres de cárie. Os dentes foram divididos em seis grupos experimentais, de acordo com: superfície do esmalte (Esmalte Sadio e Fluorótico); tratamento de superfície (Condicionamento com ácido fosfórico a 37% [ER]; Hipoclorito de sódio a 5,2% + ácido fosfórico [NaOCl + ER]; e jateamento + ácido fosfórico [Jateamento + ER]). Após armazenamento em água destilada (37^o^C/24 h), os espécimes foram testados a 1 mm/min até a falha (SBS). As interfaces esmalte-cimento foram avaliadas para GC usando espectroscopia micro-Raman. O padrão do condicionamento do esmalte foi avaliado sob microscopia eletrônica de varredura. Os dados da SBS e GC foram analisados utilizando ANOVA dois fatores e teste de Tukey (α= 0,05). O padrão de condicionamento do esmalte foi avaliado apenas qualitativamente.

**Resultados:**

Para o esmalte sadio, o ER apresentou os maiores valores de SBS, quando comparado aos grupos NaOCl + ER e Jateamento + ER (*p*< 0,01). Em relação ao GC, apenas a média do grupo NaOCl + ER foi comprometida significativamente em comparação aos outros grupos (*p*= 0,002). Para o esmalte fluorótico, Jateamento + ER aumentou significativamente a média dos valores de SBS, em comparação com o grupo ER (*p*= 0,01) e nenhuma mudança significativa foi observada no GC (*p*> 0,52).

**Conclusões:**

A aplicação de NaOCl ou jateamento associado ao ácido fosfórico melhorou a SBS dos braquetes no esmalte fluorótico sem comprometer o GC do cimento resinoso, com a melhoria do condicionamento interprismático do esmalte.

## INTRODUCTION

The success of the orthodontic treatment using fixed appliances depends substantially on the enamel-brackets bonding.^1^ Unfortunately, bracket bonding failure during the course of orthodontic treatment is a common complication in daily practice,^2^ and it is associated with emergency appointments, thus prolonging treatment time and promoting discomfort to the patients. 

Although bracket bonding failure can occur in sound enamel, a worse adhesion is expected when bonding to fluorotic enamel. Fluorotic enamel is more porous and hypomineralized, with often smaller crystallites.^3^ ^,^ ^4^ Additionally, it has been reported that the mineralized surface layer contains hydrohyapatite, fluoridated-hydroxyapatite and fluorapatite crystals more acid resistant,^5^ which a significantly higher protein content as compared to normal enamel,^3^ compromising the adequate enamel-bracket bonding. 

Thus, alternative treatments to increase the bracket retention in fluorotic teeth are suggested.^6^ One of them is to increase enamel surface roughness applying an intraoral sandblasting^7^ with aluminum oxide particles propelled by air pressure, promoting microscopic conditioning.^8^ ^,^ ^9^ Other alternative is to apply a deproteinization agent, due to higher amount of organic matrix in fluorotic enamel.^3^ Sodium hypochlorite (NaOCl) solution removes the excess of protein content^10^ and may be a possible strategy to optimize adhesion by removing organic elements of the enamel structure and the biofilm.^11^

Moreover, the conversion of monomer into polymer plays an important role in successful enamel-brackets bonding.^12^ ^,^ ^13^ The conversion degree of orthodontics resin cement was previously reported; however, the authors^12^ did not evaluate the conversion degree into the adhesive interface especially after sandblasting or deproteinization treatment in fluorotic enamel.

Additionally, both alternatives (sandblasting or deproteinization agent) were not compared in the same study^6^ ^,^ ^14^ ^,^ ^15^ in fluorotic enamel. Thus, the aim of the present study was to compare the effectiveness of pretreatment using deproteinization with NaOCl or sandblasting on shear bond strength; *in situ* conversion degree of brackets in fluorotic enamel, and enamel etching pattern were also compared 

## MATERIAL AND METHODS

### Tooth selection and specimen preparation

Diagnosis of dental fluorosis was made according to the severity using the Thylstrup and Fejerskov index (TFI).^16^ Previously to selection of teeth, two examiners were submitted to training and calibration procedure according to Ermis et al.^17^ A total of 90 non-carious human maxillary premolars were used. Forty-five fluorosed teeth with TFI score of 4 and forty-five with TFI of 0 (without fluorosis), were obtained. The teeth were collected after obtaining the patients’ informed consent under a protocol approved by the Ethics Committee Review Board of the *Universidade Estadual de Ponta Grossa* (2.522.293). The teeth were disinfected in 0.5% chloramine, stored in distilled water, and used within six months of extraction. 

### Experimental design and sample size calculation

Ninety teeth (45 TFI = 0 and 45 TFI = 4) were then assigned to six experimental groups (n = 15 per group; 10 to shear bond strength; 4 to *in situ* conversion degree, and 1 to enamel etching pattern) according to the combination of the independent variables: enamel surface (sound or fluorotic enamel); surface treatment (regular etch with 37% phosphoric acid [RE]; 5.2% sodium hypoclorite [Fórmula & Ação, São Paulo/SP, Brazil] + phosphoric acid [NaOCl + RE]; and sandblasting [RONDOflex Plus, Kavo Kerr, Joinville/SC, Brazil] + phosphoric acid [Sandblasting + RE]). 

For establishing the sample size, the bond strength values of metallic brackets to fluorotic enamel were considered. According to previous literature, mean and standard deviation of metallic brackets to fluorotic enamel was 11.0 ± 3.1.^6^ ^,^ ^14^ ^,^ ^18^ Using an α of 0.05, a power of 90% and a two-sided test, the minimal sample size was 10 teeth in each group in order to detect a difference of 5 MPa among the tested groups.

### Bonding procedures

For shear bond strength (SBS) test, the roots of the 60 teeth were centrally embedded in a polyvinyl chloride tube (10 mm high x 13 mm diameter) using a chemically cured acrylic resin (Jet Clássico, São Paulo/SP, Brazil) until two-thirds of the root, with the labial surfaces parallel to the mold base so that they would be parallel to the force during the bond test. The buccal surface of each tooth was positioned perpendicularly to the base and the buccal surfaces of the teeth were cleaned and polished with oil- and fluoride-free fine pumice using a slow-speed handpiece, then rinsed with water and dried.

All step-by-step bonding procedures are described in Table 1 according to the respective groups. For all groups, the bracket bonding was made with Orthocem resin cement (FGM Dental Products, Joinville/SC, Brazil), according to the experimental groups (Table 1). After the surface pretreatment, the enamel surface was etched with 37% phosphoric acid (Condac 37, FGM Dental Products, Joinville/SC, Brazil) for 30 s, rinsing for 15 s and air-dry for 30 s. A small amount of the bonding resin was applied to the bracket (BioQuick^?^, Forestadent^?^, Pforzheim, Germany) and positioned on the flat surface and pressed. The excess of the resin cement was removed with a sharp explorer and light-curing was performed using a LED light-curing unit set at 1000 mW/cm^2^(Valo, Ultradent Products Inc, South Jordan, UT, USA). A radiometer (Demetron L.E.D. radiometer, Kerr, Victoria, Australia) was used to check the light intensity every five specimens. 

**Table 1:** Resin cement (batch number), composition, groups, and application mode.

Resin cement (batch number)	Composition	Groups	Application mode (*)
Orthocem FGM Dental Products (# 141217)	Resin cement: BisGMA, TEGDMA, methacrylic phosphatized monomers, stabilizer, CQ, co-initiators, silicon dioxide nanometric loading.	Sound and fluorotic enamel (RE)	1. Apply 37% phosphoric acid (Condac 37) for 30 s 2. Rinse for 30 s and air-dry 3. Apply small amount of resin cement onto the base of the bracket and set it on position 4. Remove the excess 5. Light-cure for 20 s at 1200 mW/cm^2^ for each margin.
		Sound and fluorotic enamel + NaOCl 5.2% (NaOCl + RE)	1. Actively apply 5.2% NaOCl for 1 min 2. Apply 37% phosphoric acid (Condac 37) for 30 s 3. Rinse for 30 s and air-dry 4. Apply small amount of resin cement onto the base of the bracket and set it on position 5. Remove the excess 6. Light-cure for 20 s at 1200 mW/cm^2^ for each margin.
		Sound enamel and fluorotic enamel + sandblasting (Sandblasting + RE)	1. Sandblasting with 27-µm aluminum oxide at 80 psi for 20 s at 5 mm from labial surface at a 90° angle. 2. Apply 37% phosphoric acid (Condac 37) for 30 s 3. Rinse for 30 s and air-dry 4. Apply small amount of resin cement onto the base of the bracket and set it on position 5. Remove the excess 6. Light-cure for 20 s at 1200 mW/cm^2^ for each margin.

*The materials were applied according to the recommendations of their respective manufacturers.

Bis-GMA = bisphenolglycidyl methacrylate; TEGDMA = triethylenelglycidyl methacrylate; CQ = camphorquinone.

### Shear bond strength testing

After storage in distilled water for 24 hours at 37°C, the specimens were attached to a shear-testing device (Odeme Biotechnology, Joaçaba/SC, Brazil) and tested in a universal testing machine (Kratos IKCL 3-USB, Kratos Equipamentos Industriais Ltda, Cotia/SP, Brazil) with a 500-N load cell. Each specimen was positioned in the universal testing machine and a chisel tip was placed onto the bracket-enamel interface. The setup was maintained in alignment (resin cement-enamel interface, the chisel, and the center of the load cell) to ensure the correct orientation of the shear forces. The crosshead speed in the compressive mode was set at 1 mm/min until failure. 

The SBS values (MPa) were calculated by dividing the load at failure by the surface area (mm^2^). After testing, the specimens were examined in an optical microscope (SZH-131, Olympus Ltd, Tokyo, Japan) at 10x magnification, to define the adhesive remnant index (ARI) adhered to the tooth and bracket after bracket debonding.^19^ All teeth were analyzed by the same evaluator. The ARI modified was used to classify the failure modes: score 0 = no resin cement left on the tooth; score 1 = less than half of resin cement left on the tooth; score 2 = more than half of resin cement left on the tooth; score 3 = all resin cement left on the tooth with distinct impression of the bracket base. ^19^

### In situ conversion degree (CD)

Twelve sound enamel teeth and twelve fluorotic enamel teeth were used in this topic. The roots of the teeth were removed by sectioning at the cementoenamel junction. The enamel surface was treated and resin cement build-ups were constructed on the bonded enamel using the same protocols described for the SBS test. After storage of the restored teeth in distilled water at 37°C for 24 hours, the resin cement-enamel specimens were longitudinally sectioned across the bonded interface with a low-speed diamond saw (Isomet, Buehler Ltd, Lake Bluff, IL, USA) to obtain two resin-enamel slices.

The resin cement-enamel slices were wet polished with 1500-, 2000- and 2500-grit SiC paper for 15 seconds each. Then they were ultrasonically cleaned for 20 minutes in distilled water and stored in water for 24 hours at 37°C. The micro-Raman equipment (XploRA ONE^TM^ Raman microscope, HORIBA Scientific, New Jersey, NY, USA) was first calibrated for zero and then for coefficient values using a silicon sample. The samples were analyzed using a 638-nm diode laser through an x100/0.9 NA air objective. The Raman signal was acquired using a 600-lines/mm grafting centered between 600 and 1800 cm^-1^, and the employed parameters were 100 mW, spatial resolution of 3 µm, spectral resolution of 5 cm^-1^, accumulation time of 30 s, with 5 co-additions.

Spectra were taken at the resin cement-enamel adhesive interface at three dissolver sites for each specimen. Spectra of uncured resin cement were taken as references. The ratio of double-bond content of monomer to polymer in the adhesive was calculated according to the following formula: DC (%) = (1- [R cured / R uncured]) × 100, where R is the ratio of aliphatic and aromatic peak intensities at 1639 cm^-1^ and 1609 cm^-1^ in cured and uncured resin cement.

### Enamel etching pattern

The enamel-etching pattern was qualitative evaluated on the enamel surface under scanning electron microscope (MIRA TESCAN, Shimadzu, Tokyo, Japan). For this purpose, sound enamel teeth (n = 3) and fluorotic enamel teeth (n = 3) were sectioned in the diagonals across the long axis of the tooth with a water-cooled low-speed diamond saw (Isomet 1000) in order to obtain four enamel specimens.^20^ The enamel specimens were conditioned according to the following groups:

1. Regular etch with 37% phosphoric acid (RE);
2. NaOCl + phosphoric acid (NaOCl + RE);
3. Sandblasting + phosphoric acid (Sandblasting + RE).

The surfaces were then rinsed off with tap water for 30 s and air dried with an air spray for 5 s. All specimens were dried and dehydrated in a desiccator for 12 hours, and the conditioned enamel surfaces were sputter coated with gold/palladium in a vacuum evaporator (SCD 050, Balzers, Schaan, Liechtenstein). The entire surface of treated enamel was examined under a scanning electron microscope (MIRA TESCAN, Shimadzu, Tokyo, Japan). Photomicrographs of representative surface areas were taken at 5000x magnification.

### Statistical analysis

After evaluation of the normality by the Shapiro-Wilk test and homoscedasticity of the variances by the Bartlett test (not shown data), data from SBS and *in situ* CD values were analyzed using two-way ANOVA (enamel surface *vs* surface treatment) and Tukey *post-hoc* test at a level of significance of 5%. The enamel etching pattern was evaluated only qualitatively.

## RESULTS

### Shear bond strength testing

The ARI of all groups showed a higher variability between failures scores (Table 2). The RE group showed higher presence of scores 2 and 3 (90%) in sound enamel. On the other side, in fluorotic enamel, 90% of the failures were scored as 0 or 1. In sound enamel, the failure pattern to deproteinization (NaOCl + RE) showed scores 1 and 2 (50% each one) for sound enamel, and scores 1 and 3 for fluorotic enamel. When Sandblasting + RE group was evaluated, the failure pattern was 90% scores 0 for sound enamel and 90% scores 2 and 3 for fluorotic enamel.

**Table 2:** Percentage of Adhesive Remnant Index (ARI) according to the each score of the different experimental groups

Groups	Sound enamel (ARI)				Fluorotic enamel (ARI)			
	0	1	2	3	0	1	2	3
RE	--	10	40	50	80	20	--	--
NaOCl + ER	--	50	50	--	--	20	30	50
Sandblasting + RE	90	10	--	--	--	10	40	50

(*) ARI scores: score 0 = no resin cement left on the tooth; score 1 = less than half of resin cement left on the tooth; score 2 = more than half of resin cement left on the tooth; score 3 = all resin cement left on the tooth, with distinct impression of the bracket base.

Regarding to two-way ANOVA test of SBS values, the cross-product interaction enamel surface *vs* surface treatment was statistically significant (*p*= 0.01,Table 3). The application of RE group in sound enamel showed the highest and statistically significant SBS value, when compared to all groups (*p*= 0.01, Table 3). For sound enamel, both alternative treatment (NaOCl + RE and Sandblasting + RE) significant decrease the SBS values (*p*= 0.01, Table 3). For fluorotic enamel, the application of NaOCl + RE, as well as Sandblasting + RE, increased the SBS values, but only significantly when Sandblasting + RE was compared with ER group (*p*= 0.01, Table 3). 

**Table 3:** Mean and standard deviations of the shear bond strength (MPa) values of the different experimental groups

	Sound enamel	Fluorotic enamel
RE	17.3 ± 2.1^a^	9.7 ± 2.1^c^
NaOCl + ER	11.8 ± 3.2^b^	11.7 ± 2.8^b.c^
Sandblasting + RE	12.2 ± 2.7^b^	12.7 ± 2.1^b^

(*) Different letters indicate means statistically different (Two-way ANOVA and Tukey test; p = 0.01).

### In situ conversion degree (CD)

Regarding to two-way ANOVA test of CD values, the cross-product interaction enamel surface*vs*surface treatment was statistically significant (*p*= 0.002,Table 4). The application of RE in sound enamel showed higher and statistically similar CD value when compared to Sandblasting + RE (*p*= 0.32,Table 4). For sound enamel, the application of NaOCl + RE significantly decreased the CD values (*p*= 0.002,Table 4). For fluorotic enamel, the application of NaOCl + RE or Sandblasting + RE did not significantly change the CD, in comparison with RE group (*p*> 0.52, Table 4). 

**Table 4:** Mean and standard deviations of the in situ conversion degree (%) values of the different experimental groups

	Sound enamel	Fluorotic enamel
RE	70.3 ± 2.8^a^	58.0 ± 2.0^b^
NaOCl + ER	61.2 ± 1.7^b^	57.1 ± 1.9^b^
Sandblasting + RE	68.6 ± 2.1^a^	57.3 ± 2.4^b^

(*) Different letters indicate means statistically different (Two-way ANOVA and Tukey test; p = 0.002).

### Enamel etching pattern

**Figure 1:**
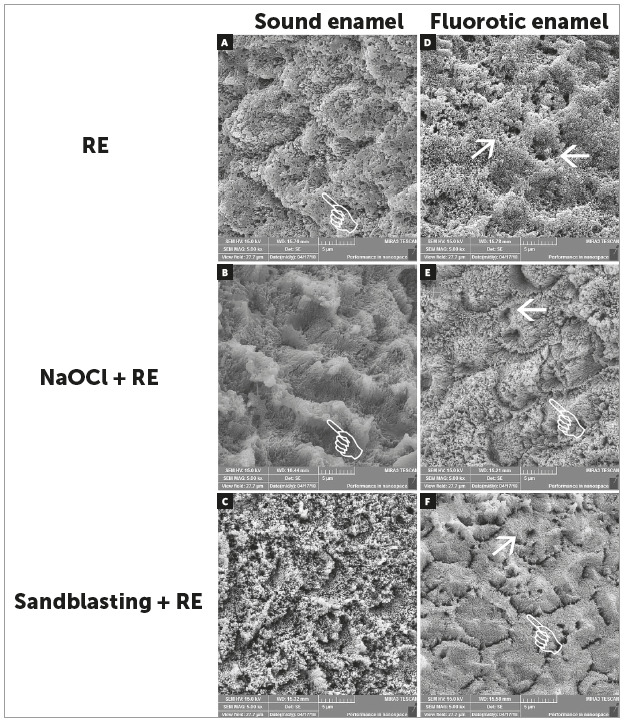
Representative morphology of sound (A, B, C) and fluorotic enamel (D, E, F) after different treatment. RE resulted in a best-defined etching pattern (white hand, in A). NaOCl significantly changed the quality of the etching pattern and prism periphery (white hands, in B). Sandblasting promoted a significant structural destruction, with total prism core dissolution (C). Fluorotic enamel showed an increase of the porous number (white arrows in D to F). Better signs of the interprismatic conditioning were observed after treatment in fluorotic enamel (white hands, in E and F). Prism peripheries have been only partially removed (white hand, in F), becoming more pronounced, leaving the prism cores relatively intact (white arrow, in F).

After qualitative evaluation in sound enamel, the RE promoted a deepest and most organized etching pattern, with a presence of the prism core and an intact prism periphery. After NaOCl + RE treatment, a higher dissolution of the mineral content and prism periphery was observed. After Sandblasting + RE, an increase of the surface modification and dissolution on the prism core, with a significant destruction structural of the aprismatic area, was found, when compared with other groups (Fig 1). 

According to qualitative evaluation of fluorotic enamel, an increase of the micro-irregularity and porosity was observed independently of the treatment. NaOCl + RE and Sandblasting + RE groups increased the dissolution of prism core with improving of interprismatic conditioning (Fig 1). When Sandblasting + RE was applied, the prism peripheries have been partially removed, becoming more pronounced, leaving the prism cores relatively intact (Fig 1).

## DISCUSSION

The results of the present study showed that the application of only phosphoric acid showed the highest bond strength to sound enamel, when compared to fluorotic enamel. It is known that structural differences between normal and fluorotic enamel teeth can influence the bond strength.^17^ ^,^ ^21^ Dental fluorosis is characterized by a hypermineralized layer, with the presence of fluorapatite in the outer enamel surface more crystalline and stable, making it resists dissolution in acid-etchant.^3^ Thus, these morphological alterations could have promoted the reduction of the bond strength to fluorotic enamel. Additionally, a higher number of ARI scores 0 and 1 was observed in fluorotic enamel, showing the lower interaction with the fluorotic enamel surface in general. On the other side, in the sound enamel, more than 90% were ARI scores 2 and 3, showing intense interaction with surface of sound enamel. It is known that a direct correlation between higher ARI score and higher bond strength is expected.^22^

Some reports have suggested that the application of NaOCl before etching eliminates the organic substances from the enamel surface, and this may increase the bond strength to enamel because it results in an increase in the total conditioning area.^11^ ^,^ ^23^ On the other hand, several studies have shown that de deproteinization with NaOCl decreased the bond strength of metallic bracket due to incapacity of improving the quality of the decalcification pattern.^24^ ^,^ ^25^ The results of the present study are in accordance to Trindade et al,^24^ in which the application of NaOCl significantly decreased the bond strength to sound enamel. Additionally, it is known that NaOCl forms reactive free radicals and can inhibit the adequate polymerization.^26^ ^,^ ^27^ These reactive residual free radicals compete with the propagating vinyl free radicals generated during light activation, resulting in premature chain termination and incomplete polymerization.^26^ Thus, the authors of the present study also speculates that the presence of these residual radicals on the sound enamel decreases the conversion degree inside the enamel-resin cement interface and consequently promotes a reduction of the bond strength values.

However, when NaOCl was applied on fluorotic enamel, an increase in the bond strength values was observed. It is known that fluorotic enamel contains significantly higher protein content.^3^ Thus, it can be hypothesized that the sodium hypochlorite reacted with the higher protein content present on the fluorotic enamel, ^3^ generating less reactive residual free radicals to inhibit the adequate polymerization without compromising the conversion degree. Unfortunately, these results cannot be compared with previously literature, mainly because, to the extent of author’s knowledge, this is the first study that evaluated the *in situ* conversion degree inside the enamel-resin cement-bracket interface. Therefore, future studies are needed to prove this hypothesis.

Regarding the use of sandblasting, controversial results for bond strength values were observed in the literature when sound and fluorotic teeth where compared.^9^ ^,^ ^14^ ^,^ ^18^ ^,^ ^28^ ^-^ ^30^ It has been suggested that sandblasting of enamel in association with phosphoric acid removes oxides and contaminants from teeth surface, increasing the total energy surface and roughness.^9^ This effect could be partially showed when observing the microscopy evaluation for sound and fluorotic enamel. It could be seen a significant increase of the roughness and porosity existing in both enamel substrates after sandblasting and phosphoric acid, when compared with only phosphoric acid.

However, for sound enamel, the sandblasting decreased the bond strength values when compared to RE group. On the other hand, in fluorotic enamel, a significant increase in the bond strength values occurred when sandblasting was applied. These different results are also showed in the evaluation of the ARI score. A higher number of scores 2 and 3 (90%) for sandblasting + RE occurred in the fluorotic enamel, meaning that at least more than half of resin cement was left on the fluorotic enamel surface. However, when the sandblasting + RE was applied in the sound enamel, the ARI scores were predominantly 0 and 1, meaning lower interaction with the sound enamel surface. 

Based on these findings, it is inferred that a good interaction between sound enamel and resin cement used after sandblasting application in sound enamel does not occur. It is unclear for the authors of the present study what is the reason for the decrease on bond strength values in sound enamel. However, although exists a common sense that sandblasting has a positive effect in the sound enamel, a recently published systematic review and meta-analysis of *in vitro* studies showed that the sandblasting did not increase the bond strength values of orthodontic brackets.^9^ However, the extrapolation of these findings is limited because the conclusion was only supported by two *in vitro* studies.^9^ The lack of standardizing in methodological approaches for different studies could be the reason for the lower number of *in vitro* studies evaluated in the Baumgartner’ study.^9^ Therefore, future better designs and controlled *in vitro* studies are needed to evaluate the effect of these variables in sound enamel.

Surprising, in fluorotic enamel a significant increase in the bond strength values when sandblasting was applied occurred. These findings are also controversial.^14^ ^,^ ^28^ As fluorotic enamel is less reactive than sound enamel, it is reasonable to speculate that the microporosities of the fluorotic enamel surface after the application of sandblasting is improved, thus increasing the bonding area^14^ ^,^ ^31^ and, consequently, producing a significant increase of the bond strength values,^14^ ^,^ ^28^ when compared to only phosphoric acid applied in fluorotic enamel. 

It is important to mention the possible limitations in the present study. The results of the present study are based on the immediate results, without any aging method. Commonly, thermocycling is the common method used to evaluate bond durability^32^ ^-^ ^34^ and simulate the thermal changes that occur in the oral environment.^34^ However, the varied number of cycles, the choice of temperature, time conditions, and intervals between baths hinder comparison of the results.^6^ ^,^ ^13^ ^,^ ^34^ Therefore, further studies should be conducted to investigate if the NaOCl and sandblasting can preserve the resin cement-fluorotic enamel interface from degradation in longer periods of time. 

Thus, the results of the present study suggest that the alternative surface treatment evaluated (NaOCl or sandblasting) improve the bond strength on fluorotic enamel without compromising the conversion degree of the resin cement used. 

## CONCLUSIONS

The application of NaOCl or sandblasting associated with phosphoric acid improved the bonding of the metallic brackets in fluorotic enamel without compromising the *in situ* conversion degree of the resin cement. The treatment compromise the bond strength to sound enamel.
